# Bioengineered extracellular vesicle-loaded bioscaffolds for therapeutic applications in regenerative medicine

**DOI:** 10.20517/evcna.2021.10

**Published:** 2021-06-30

**Authors:** Sabrina Lazar, Sirjan Mor, Jianing Chen, Dake Hao, Aijun Wang

**Affiliations:** 1 Department of Surgery, University of California, Davis School of Medicine, Sacramento, CA 95817, USA.; 2 Institute for Pediatric Regenerative Medicine, Shriners Hospitals for Children, Sacramento, CA 95817, USA.; 3 Department of Biomedical Engineering, UC Davis, Davis, CA 95616, USA.

**Keywords:** Extracellular vesicles, biomaterials, bioscaffolds, EV scaffolds, EV therapeutics, drug delivery, bioengineering

## Abstract

Extracellular vesicle (EV)-based technologies represent a new advancement for disease treatment. EVs can be administered systemically, injected into the injury site directly, or applied locally in conjunction with bioengineered implantable scaffolds. Matrix-bound vesicles (MBVs), a special class of vesicles localized in association with the extracellular matrix (ECM), have been identified as critical bioactive factors and shown to mediate significant regenerative functions of ECM scaffolds. Loading EVs onto bioscaffolds to mimic the MBV-ECM complex has been shown superior to EV bolus injection in recent *in vivo* studies, such as in providing enhanced tissue regeneration, EV retention rates, and healing efficacy. Different types of natural biomaterials, synthetic polymers, and ceramics have been developed for EV loading, and these EV functionalized biomaterials have been applied in different areas for disease treatment. The EV functionalized scaffolds can be designed to be biodegradable, off-the-shelf biomaterials as a delivery vehicle for EVs. Overall, the bioengineered EV-loaded bioscaffolds represent a promising approach for cell-free treatment in clinical applications.

Extracellular vesicles (EVs) are nanoparticles of various sizes secreted by all cells that can act as cell-cell communicators^[1]^ because they contain RNAs, proteins and lipids to facilitate intercellular communications^[2]^. Moreover, EVs can make an efficient drug delivery system because they carry the intrinsic ability to cross cellular/tissue barriers such as the blood-brain barrier^[3]^. Hence, EVs have emerged as a promising strategy in regenerative medicine. The most common mode of EV delivery for tissue repair is systemic intravenous injection of free EVs or local injection directly into sites of injury, which unfortunately may lead to rapid clearance of EVs. Many different approaches have been used to optimize the delivery strategy of EVs, such as increasing the injection dose, surface modification for targeted delivery, or encasing EVs in a biomaterial matrix^[1,4,5]^. Since EVs arise from cellular paracrine secretions and carry cellular membrane compositions, they often interact with the surrounding extracellular matrix (ECM) environment *in vivo*. In addition, matrix-bound vesicles are identified as an integral and functional component of ECM biomaterial scaffolds mediating significant regenerative functions^[6,7]^. Therefore, integrating EVs and ECM-mimicking biomaterials to mimic the native EV-ECM complexes provides great potential for preservation and sustained delivery of EVs for regenerative medicine applications [Figure 1]^[8]^. In this commentary, we will outline the recent advances in the administration of EV-functionalized biomaterials and discuss future challenges in this field.

Scaffolds have been widely used to provide physical support for the loading of EVs at injury sites. For example, bioscaffolds can deliver mesenchymal stem/stromal cell derived EVs (MSC-EVs) at specific areas to repair peripheral nerve injury, epidural fibrosis and incisional hernia^[1]^. EV-loaded scaffolds present an opportunity to advance drug delivery. In addition, EVs can be engineered by genetically modifying EV-producing parent cells, fusing targeting proteins or aptamers to EV surfaces, or altering internal cargo^[3,9]^. The EV-scaffolds can be directly applied to disease areas, serving as sustained release devices to extend the EVs’ retention and prevent mass diffusion away from the site or enzymatic digestion. This approach is more optimal than EV or drug intravenous injection, which could lead to off-target EV accumulation^[10]^. In addition, EV/scaffold complexes can be developed using a variety of biomaterials and can be optimized for disease-specific or tissue-specific applications.

In order to be safe and effective, bioscaffolds must fulfill criteria such as biocompatibility, degradability and the necessary mechanical properties^[1]^. Natural biomaterials such as collagen, hyaluronan, and decellularized ECM materials can be used^[1]^. These materials provide excellent specificity for cell/EV surface receptors, however there is more heterogeneity in natural biomaterials due to intrinsic variations from their biological sources^[11,12]^. Synthetic materials such as FDA-approved polylactide-*co*-glycolide and beta-tricalcium phosphate^[1]^ can also be used. Synthetic materials are generally cheaper and more homogenous than natural materials in their biological properties, and they can be modified to exert specific biological activities. Natural materials and synthetic materials can also be combined or chemically modified to be used as hybrid biomaterials^[1]^.

To imbue scaffolds with biological activity, EVs can be chemically conjugated to them using targeted proteins or ligands. For example, an integrin α4β1 ligand LLP2A was found to bind strongly to placenta mesenchymal stem cell derived EVs (PMSC-EVs). LLP2A immobilized to a polymer scaffold via Click chemistry^[8]^ can be used to specifically load PMSC-EVs onto the scaffold and the EV-loaded scaffold increased angiogenesis and vascularization in an *ex vivo* aortic ring sprouting assay^[8]^. Others have leveraged ECM-related proteins to increase EV immobilization by coating scaffolds with fibronectin^[13]^. On a decellularized bone matrix scaffold coated with fibronectin, bone marrow mesenchymal stem cell derived EVs promoted bone regeneration and angiogenesis *in vivo*^[13]^. When applied to injury sites, the EVs on the scaffold may be protected and released in a sustained manner from the scaffold and communicate with endogenous cells and extracellular components to participate in the remodeling process.

Using bioscaffolds to immobilize and deliver EVs has been shown to be more effective than injecting free EVs when applied *in vivo*. For example, compared to bolus EV injections, EV-functionalized polyethylene glycol hydrogels significantly enhanced liver regeneration by attenuating inflammation and apoptosis in a rat model of chronic liver fibrosis^[14]^. In a myocardial infarction rat model, EV-loaded peptide hydrogels were superior to EV bolus injection in increasing angiogenesis and reducing inflammation^[15]^. Bioscaffold-based EV delivery may be more advantageous than traditional EV injections in improving retention and targeted delivery of EVs to the site of injury^[10,12,16]^.

EVs as a special biological component provide more possibilities to functionalize scaffold materials with biological functions. By integrating biochemistry and bioengineering principles, EV bioscaffold products have shown promising therapeutic outcomes in numerous medical studies, such as wound healing, tissue regeneration, vascularization, and angiogenesis [Figure 1]^[8,13,17]^. In addition, appropriate EV delivery systems have shown obvious advantages for further enhancing the function of EV modified bioscaffolds^[15,18]^. Therefore, future research may focus on further refinement of EV modified scaffolds, such as the loading and release mechanisms, the loading density and release profile, storage stability, and safety must be fully characterized before clinical applications. Scaffold-based EV delivery is becoming a promising cell-free therapeutic approach for tissue regeneration and clinical applications.

## Figures and Tables

**Figure 1.**
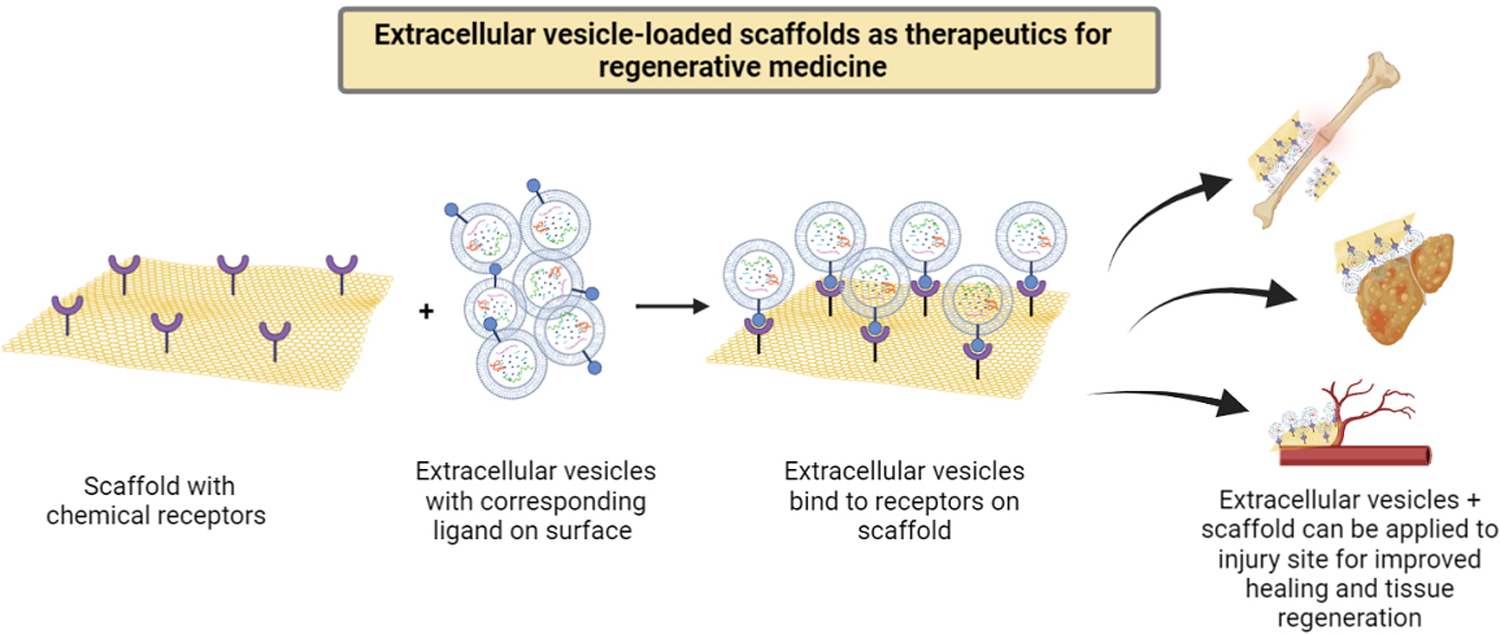
Extracellular vesicles (EVs) and scaffolds can be designed to bind specifically to each other and be applied together onto injury or disease sites. For example, EV bioscaffolds provide localized delivery and sustained therapeutic EV release, and can be applied for wound healing, tissue regeneration, neovascularization, or angiogenesis *in vivo*.
